# COVID-19 Amplifiers on Health Inequity Among the Older Populations

**DOI:** 10.3389/fpubh.2020.609695

**Published:** 2021-01-15

**Authors:** Sora Lee

**Affiliations:** School of Regulation and Global Governance, Australian National University, Canberra, ACT, Australia

**Keywords:** COVID-19, aging, health equity, social determinants, public health policy

## Abstract

The coronavirus disease 2019 (COVID-19) is affecting the population disproportionately and is continuously widening the health gap among the population. Based on some recent studies on COVID-19 and the older population, the various cascades toward health inequity have been projected. This study highlights how the COVID-19 is met by health inequity triggers, such as global trade inequality, ageist social regulations, and the existing social inequity. While those triggers are applicable to all the populations, there seems to be specific amplifiers for health inequity among the older populations. In particular, six types of amplifiers have been identified: (1) expansion of riskscape, (2) reduction of social ties, (3) uncertainty of future, (4) losing trust in institutions, (5) coping with new knowledge, and (6) straining on public spending. While the fundamental mitigating responses to health inequity among the older population is tackling existing inequalities, this study may help to shed light on emerging vulnerabilities among the older population to alleviate far-reaching consequences of COVID-19 of the identified inequity amplifiers.

## Introduction

The coronavirus disease 2019 (COVID-19) pandemic is an ongoing global health threat, with 30.6 million cases and 954,417 deaths confirmed worldwide as of 20th September 2020 (1). The impact of COVID-19 has been particularly severe for older populations. Nearly 25% of deaths due to COVID-19 have been the population group over the age of 70 (2). Furthermore, numerous cases of COVID-19 outbreak occurred in nursing homes and the devastating consequences have reached the media. One of the underestimated aspects of COVID-19 is that it is not an “equalizer” but is continuously widening the gap (3). The COVID-19 is affecting the population disproportionately, and older population groups are exhibiting higher vulnerability to COVID-19.

The widening gap of COVID-19-related health status within the older population is socially determined (4). Health inequity is defined by Whitehead (5) as differences in health that are unnecessary, avoidable, unfair, and unjust. The pandemic adds another process to the existing interplay between their health status and social vulnerability, such as access to healthcare, housing, income inequality, and cultural beliefs, under the COVID-19 (4). These “conditions in the places where people live, learn, work, and play that affect a wide range of health risks and outcomes” are called social determinants of health (6). COVID-19 incidence, prevalence, testing, treatment, and mortality are heavily influenced by social determinants (7). The individual social backgrounds as well as the policies, mitigation, and adaptation strategies that one is endowed with determine one's interaction with this highly transmittable disease (4, 8). Yet, how the COVID-19 is further constraining the existing social determinants and creating new social conditioning on the health among the older populations still remains elusive. This study aims to fill the gap by identifying triggers and amplifiers on social determinants, driven by COVID-19, and how they influence health inequity among the older population.

## Method

**Figure 1:**
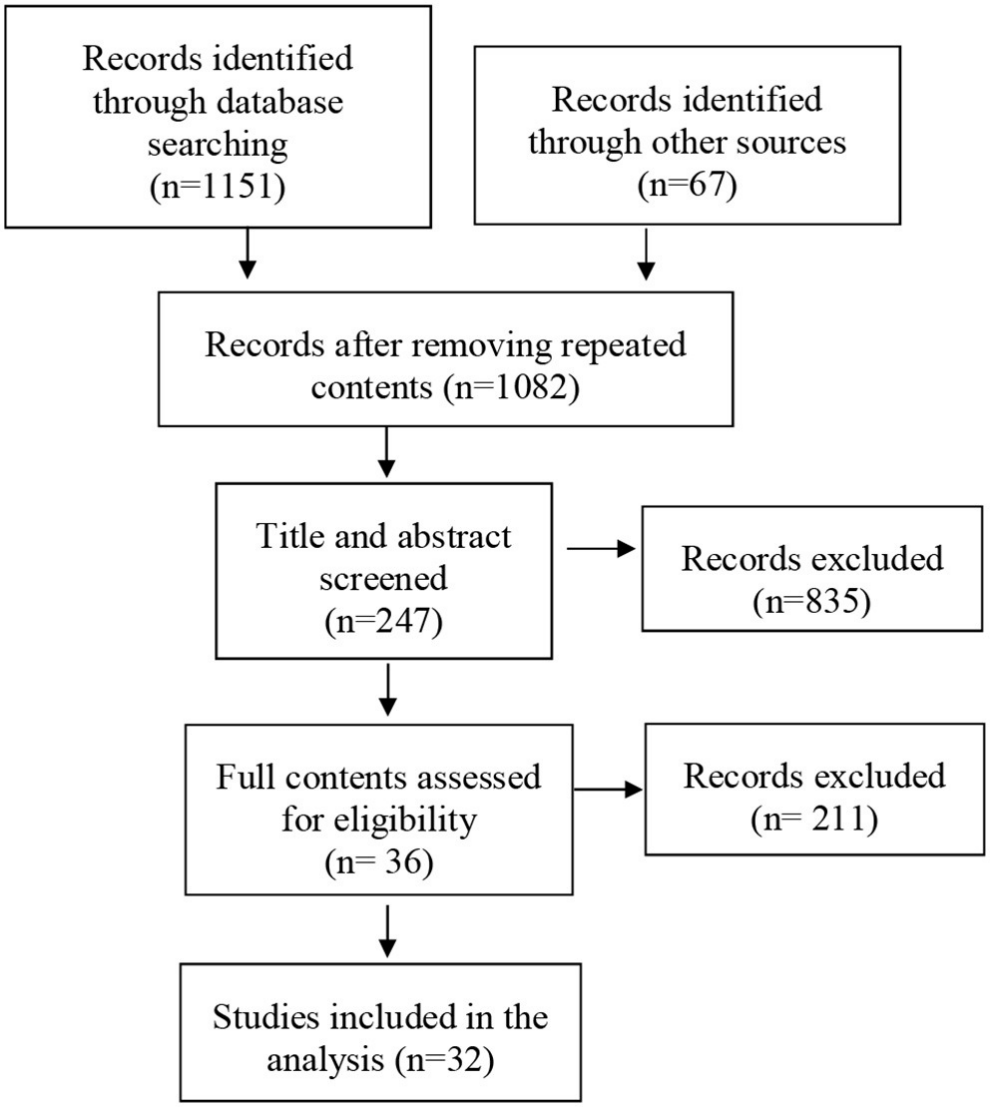
Selection process for the scoping review. The conceptual model of COVID-19 triggers and amplifiers on health inequity among the older populations.

The conceptual model is based on an interdisciplinary literature review, incorporating the findings from journals and news articles on the older population coping during the COVID-19 with the lens of social determinant of health. The searches for peer-review literature were conducted in multiple databases: ProQuest, Web of Science, Social Sciences Citation Index, ScienceDirect, and EBSCO. The search term included COVID-19, health equity (inequity), and older population and social determinants. The timeframe of the data collection is from March 1st to October 30th this year. Initially, total of 1,218 articles came up. The contents that focus on the COVID-19-triggered health equity impact, specifically for the older population, were selected for the purpose of this study. Clinical studies on older populations that investigate the pathogen pathways to human systems without implications of the wider context of social determinants or belong in the technical subfields are excluded for this review. After removing duplicates and content screening, 36 articles were identified, of which 32 are included in the analysis. The Figure 1 illustrates the review process explained here.

Once the author conducted the review, three distinct macro-level triggers were categorized. The triggers did not influence all population cohorts equally. Certain factors were interacting with the older cohorts of the population through their social determinants of health inequity. Then emerged a new layer on social determinants termed here as “amplifiers,” denoting a type of determinants that amplifies one's existing inequalities, potentially dampening the health inequity among the older population. Six different amplifiers were identified to highlight the new emerging patterns of health inequity among the older population due to the COVID-19.

Then follows intermediate pathways. For instance, one's chronological-age-based limited employment opportunity due to COVID-19 can put him or her into financial difficulty, which makes him dependent on inter-family resource transfers. This not only puts an excessive care burden but may also delay necessary medical treatments or procedures. Individuals with limited networks or less education background may have limitations to navigate the information necessary for the coping with COVID-19, further isolating individuals with unnecessary phobias or potentially dangerous self-medications. The list is not exhaustive and their impacts are not isolated. The conceptual model is generated to advance discussions regarding the complex interplay of COVID-19 triggers, amplifiers, and individual-level impact pathways. Thus, the study can potentially guide the counter-mechanisms for adequate policies and programs tailored for the health equity among the older populations under COVID-19.

## COVID-19 Triggers on Health Inequity

COVID-19 dampened a number of pre-existing conditions that could act as significant catalyst for population health inequity, defined here as COVID-19 health inequity triggers. This study identifies three prominent COVID-19 triggers for the dampening of health inequity. The first trigger is globally stratified essential resources for COVID-19. The pandemic has been generating inequality between countries and, if not rectified, it may potentially pose a severe future health threat such as the inequitable distribution of vaccines. The second trigger is the ageist social regulations and public responses to protect the older population. The socio-political context and various stakeholders in societies shape how society is moving toward solidarity and not toward ageism. Finally, pre-existing health inequity of a society may add a thick layer of constraint in mitigating and adapting to the COVID-19 threat.

### Global Stratification on Essential Resources for COVID-19

One of the important pillars for promoting health equity is open, transparent, and equitable global trade on essential goods (9). An immediate concern would be on ensuring the global supply of medical supplies necessary for COVID-19 responses. Export bans can be detrimental for countries without sufficient production capacity (10). Limits on the mobility of flights and people and lockdowns affect the trade processes, which in turn increases costs and time. Tariffs can impose delays in international trade, as well as increasing prices (10). While several countries maintain tariffs of up to 10% on COVID test kits (11), a number of countries are removing tariffs on essential medical goods (9). Expedited certification procedures may enhance mask production by allowing new companies to meet global demand.

However, trade facilitation is not enough to ensure an equitable distribution of essential resources for COVID-19. With the number of vaccines under clinical trials, there has been a call for an equitable global distribution infrastructure for vaccines (12). If there is no measure to alleviate financing on vaccines, the low-income countries may not be able to meet the costs of attaining the new COVID-19 vaccines (12). We need diverse cooperative financing options between developing and developed countries to ensure an equitable distribution of vaccines. Global distribution of essential resources can be an important trigger in dampening inequity between countries. In times of pandemic, we need “sympathetic hands” than “invisible hands.”

Inequity and uncertainty in international trade result in potential panic buying and hoarding of personal protective equipment for the countries affected by the limited supply. Face masks has been a pandemic icon of “coveted commodities,” exposing the political realities of international trade (13). Consumption patterns of face masks illustrate another aspect of the older population's socially determined vulnerability in digital literacy. In South Korea, young people could use this information to find masks easily, while many older adults had to take their chances with their local pharmacies (14).

### Social Measures to Protect Older Population From COVID-19

The brutal policy decisions are being made during the pandemic, which differs depending on the socio-political context of the region, country, and the city. Policymakers worldwide recognize that vulnerability of older population under the pandemic is a serious public health concern (15). Whether or not the country will be locked down or reopened, welfare provision measures among the older adults, the treatment of care workers, and the regulation change on the care facilities all significantly impacted health well-being among them. While the risk-basis measures for the older population are statistically valid (8), sustained lockdowns may become discriminatory for patients with limited resources to cope with physical isolation.

Ageist attitudes and discriminatory policies are often based on chronological stereotypes of the health and functioning of older adults (16). Despite the fact that it is hard to establish the association between the age, symptom severity, and mortality for COVID-19, underlying health conditions play a crucial role, regardless of age (17). Simply imposing an arbitrary cut-off age for lockdown would be a crude measure that aggravates the social exclusion of the older population (18–20). For instance, BHP, a resource extraction and processing company operating worldwide, has restricted older employees from working in the mines, without compensating the income loss (21).

The emphasis on the older population as a risk-prone group may impose public ageism and aggravate intergenerational tensions in societies (16). Local participatory approach can be helpful to avoid chronological age-based social measures. Governments can utilize locally based partnerships that tailor the needs and risks of the older population in the locality through the participation of local older residents, NGOs, and the public sector. Another approach is to address the ramifications of social exclusion measures in a multidimensional contexts. The cutting of ties from neighborhoods and communities, amenities and mobility, employment, social, and democratic participation needs to be restored. Furthermore, the continuous monitoring of other deteriorations in quality of life would be important. Avoiding discrimination depends not only on public policies but also on social regulation shaped by various groups, such as medical practitioners, media, and activists that are distributing information and establishing new social norms around COVID-19. Our society's older population is not an isolated group, but intricately connected with various social and familial ties that bounds them for paid and unpaid care as their duty and responsibility. Extended lockdowns or workplace exclusions would likely burden other population groups responsible for the care both physically and mentally (17).

### Pre-existing Vulnerabilities and Social Gradient Among the Older Populations

The older population in different social contexts is exposed to differential risks due to pre-existing inequalities. Although the gains in life expectancy have been extraordinary for the past three decades, they have not been distributed evenly across populations, which holds even within the same country (22). Even the advanced nations (23 OECD countries) exhibit gaps in the life expectancy of 2–3 years at age 65 between highly educated and lowly educated populations (23). The influence of socioeconomic factors on health status or outcomes at older ages is more prominent in nations with emerging economies (22). While higher income is strongly associated with better health for the emerging nations, health gaps, especially due to gender and education, were wider than in many advanced economies (23).

Some populations among the older population are at more risk than others. Older women, in particular, experience poverty in old age more frequently and those with low education (22). With income inequality rising in ~70% of OECD countries, the inequalities stemming from structural changes in the labor market is putting aging populations in a greater vulnerability (23). People who live alone experience mental distress 30% more frequently than those living with family or other counterparts, with ~50% of older population living alone reporting experiencing feeling sad or depressed. Across many G20 countries, at least 39% of people 65 and older demonstrate symptoms of mental distress, where close to 50% of women 65 and older suffer (22).

These pre-existing vulnerabilities and social gradients lead to disparity in the vulnerable population among the older adults in coping with COVID-19. In Australia, older women tend to be less financially secure and unemployed. Even if they were working, most of them have been locked out of the workforce due to COVID-19 (24). Express the concern for the rise in gender-based violence during COVID-19 based on similar projections under the Ebola and Zika outbreaks. According to an Australian study, ~31% of women killed due to domestic violence in 2019 were over 50. The isolation, anxiety, financial difficulty, lockdown, or movement restrictions due to COVID-19 put women in a riskier situation for domestic violence (25). There can be a number of amplifiers of social determinants influencing older adults differentially on the aforementioned triggers. For LMICs (low- and middle-income countries), older people face limited access to health services and support, tend to find them unaffordable, and experience ageist discrimination (26). Global stratification of resources adds to the uncertainty of the government provision in less resourceful countries, creating a country-level inequity among the older population (9). Public spending on protecting older populations and the provision of less ageist social measures is influenced by the political will and socio-cultural norms that vary depending on the societies and communities (16). Individuals with more networks and resources can afford to have the personnel and technology to cope with isolation better (3, 27). When one is not endowed with social ties, nor an occasional visit from the welfare workers, coping becomes much more challenging than those with various ties. The uncertainty of the future affects individuals more severely if one is limited in the gathering and processing of information (28). As institutional trust can be weakened by the level of civic engagement, inclusion of older people in developing governance responses may increase their trust (26). The amplifiers are discussed in the next section.

## COVID-19 Amplifiers and Their Paths to Health Inequity

Whereas, COVID-19 triggers explain the given conditions of the pandemic, amplifiers explain how those triggers are impacting the older population differentially, dampening existing inequalities among the older population. Based on the review, five COVID-19 amplifiers have been identified: expansion of riskscape, reduction of social ties, uncertainty of future, losing trust in institutions, and straining on public spending. The five COVID-19 amplifiers below is explained along with potential cascading mechanisms toward health inequity.

### Expansion of Riskscape

Riskscape is defined as “geographies of exposure and susceptibility” (29). It was first coined in environmental hazard research and its usage was expanded in diverse disciplines, including health disparities research (30). The situation for older population under the COVID-19 pandemic can be described as an expansion of riskscape on top of existing social gradient. The safe and comfortable spaces for intergenerational socializing, such as restaurants, parks, libraries, and religious spaces, have become isolated and forbidden venues for the older population (31). Everyday routines and community interactions have been disrupted (32). With limited social interaction, pre-existing neglect and violence at homes can potentially aggravate due to social distancing and lockdowns. Age-friendly services, healthcare, and community solidarity vary widely depending on their region, country, and locality, contributing to the spectrum of riskscape among the older population. Thus, local and community landscapes shaped by local government, private, and non-profit organizations' responses play critical roles for vulnerable older populations (16).

### Reduction of Social Networks

Older individual's social networks are crucial for health (33). They face COVID-19 with social isolation and loneliness (34), which are important risk factors for their mental health in old age (32). Such isolation may contribute to cascading mechanisms of disability, injury, abuse, and neglect, resulting in potential preventable and premature deaths (16, 33). It should be noted that the loss of friends and fellow community members also place high mental stress on the older survivors. Loss of in-person mourning rituals and insufficient support networks make it challenging to cope with their sadness, anxiety, anger, and fear (16). Families with intergenerational ties are subject to potential risks of transmission, in which there may be an interruption of resources and supports for the older people (35). Furthermore, there is an additional strain of resources when family members balance work and caregiving responsibilities (16). These mechanisms may strain resources across generations, contributing to intergenerational conflict in societies regarding the distribution of care and welfare resources.

### Uncertainty of Future

COVID-19 poses an inevitable uncertainty due to its unknown and evolving nature and the challenge of forecasting potential social, economic, and political impacts (28). While the uncertainty is posed to everyone, how one responds to such uncertainty depends on socio-politico-economic backgrounds, social support networks, financial situation, where one lives, and many other factors. This makes older populations extremely vulnerable in coping with uncertainty (28). The information presented may not be easy to understand; they may not attain an up-to-date latest information; perhaps they may be excluded in the decision-making process; or they may experience difficulty communicating their views (28). The stress and anxiety from the uncertainty can be a significant threat to mental health among the older population.

### Losing Trust in Institutions

Institutional trust is a form of social capital felt by citizens in their public institutions (36, 37). Developing and maintaining public trust during the pandemic is vital. Transparent, consistent, and fast responses from governments can help build the reputations and credibility of government (38). For instance, the deadly spread in recent outbreaks in nursing homes sends messages that deteriorate public trust. Caring for the older adults is considered futile, costly, and posing further threats to care workers (2). Older population may feel burdensome to other population, leading to social exclusion and loss of self-worth (34). The public trust also means losing political trust in the ruling administration. The political preference or bias may impact the scope and type of information they seek and attain. Untrustworthy governments make their information untrustworthy, resulting in potential non-compliance to the necessary adaptation and mitigation measures instructed by government authorities.

### Coping With New Knowledge and Skillsets

The information ecosystem is bombarded with contradicting and complex information on COVID-19 (39). Under these circumstances, new skill sets emerge to cope with the pandemic. Navigation practices are “the social and technical (sociotechnical) practices through which an individual traverses a metaphorical landscape of elements, interacting with a variety of touchpoints in the process of acquiring a resource(s) (e.g., specific information, a particular connection, etc) or accomplishing a needed task(s)” [(40), p. 19]. While the older population in general may not be the most apt group for this new skill sets (41), it is pointed out that their ability to cope better in terms of health literacy would be further conditioned by their resources and social capital (27). Policies addressing the digital health divide among older adults need to be widely adopted. Active promotion of online memorials, virtual funerals, and online peer support groups for the older population can help mediate the health literacy gap (42).

The lack of skill sets to attain the information on COVID-19 is not the only problem for the older population. The crisis of falsified information, called “infodemic,” is confusing and isolating the older population. The health authorities in various countries and the World Health Organization is taking the lead in stopping the spread of the misinformation around COVID-19 (43). Previous health conditions, the existing level of health literacy and bias, level of trust on the system, and social ties are expected to influence their coping with the infodemic (3). This is expected to produce patterns of social gradient among the older population.

### Straining on Public Spending

The aging population is often perceived as slowing economic growth and putting a burden on public health expenditure (44, 45). Before the pandemic, caring for the aging population is costly. Health and long-term care expenditure is expected to increase by an average of 3.9% annually between 2015 and 2030, amounting to 10.3% of GDP among 15 OECD countries by 2030 (22). COVID-19 is adding a certain degree of tension in the management of risk for different populations. According to the Centers for Disease Control and Prevention, nearly half of hospitalizations belong to age group over 55, and serious complications related to older people's pre-existing conditions are adding further straining to the public health system (46). Accessibility to healthcare may be limited due to resources; planned treatments are expected to be delayed.

## Limitation

It is true that models with certain levels of approximation of the reality, like the conceptual model presented here, run short to reflect the complexity and ambiguities of the entire picture. The illustrated mechanisms are neither exhaustive nor definite. The model can change over time, as unexpected research outputs become evident or political influence is exerted. The use of the conceptual model illustrates an approximation of the anticipated pathways of COVID-19 and aide the modalities of future interventions. In other words, it is to be used as a guide rather than a reproduction of reality. The model may be less or more applicable to a particular context, which may provide sites for future research opportunities.

## Conclusion

The conceptual model on COVID-19 amplifiers on health equity among the older population helps understand the complex, interrelated, and cascading factors on health inequity among the older population. The factors were dampening the health inequity and positing new conditions and abilities that the population is simply not used to. The model points out implications for recent ageist policy responses to COVID-19 and suggests interventions to mitigate the emerging vulnerabilities of the older population against the COVID-19, identified as amplifiers. With budget constraint, we need to develop cost-effective measures that work for the older population. Some of the mechanisms identified above may be useful in designing intervention modalities. Furthermore, as the model illustrates the anticipated pathways, further evidence-based studies can help fill the gap in the complex mechanisms of social determinants on health inequity.

## Data Availability Statement

The original contributions presented in the study are included in the article/supplementary materials, further inquiries can be directed to the corresponding author/s.

## Author Contributions

The author confirms being the sole contributor of this work and has approved it for publication.

## Conflict of Interest

The author declares that the research was conducted in the absence of any commercial or financial relationships that could be construed as a potential conflict of interest. The reviewer CJB declared a shared affiliation, though no other collaboration, with one of the authors SL to the handling Editor.
